# Social robots: a meta-analysis of learning outcomes

**DOI:** 10.3389/frobt.2025.1735198

**Published:** 2026-03-23

**Authors:** J. C. F. de Winter, D. Dodou, F. Moorlag, J. Broekens

**Affiliations:** 1 Faculty of Mechanical Engineering, Delft University of Technology, Delft, Netherlands; 2 Leiden Institute of Advanced Computer Science, Leiden University, Leiden, Netherlands

**Keywords:** cognitive skills, educational robotics, human-robot interaction, learning, social robot

## Abstract

Previous meta-analyses show that social robots aid learning but were often limited in scope or grouped diverse control conditions together. This meta-analysis examined learning outcomes, focusing on control condition type. We retrieved 146 studies (Google Scholar and reference searches) where a physical social robot was used for training cognitive skills, comprising 183 post-test effect sizes between the robot and the control group, and 372 pre-post effect sizes. Analysis of the 78 studies with control groups indicated that robots generally improved learning, most notably when compared to a no-training control group (*d* = 0.75). Comparing robots to human teachers yielded an overall positive effect (*d* = 0.31), although effect sizes varied widely. This variability was explained by the robot’s role: robots in a co-teaching capacity showed a strong positive effect (*d* = 0.88), while robots replacing the teacher showed no benefit (*d* = −0.06). LLM-based sentiment analysis indicated that papers from outside Europe received higher positivity scores when describing the robots. We conclude that the effect size is influenced by the robot implementation and the control condition chosen.

## Introduction

The integration of artificial intelligence, robotics, and educational sciences has led to the emergence of social robots within educational settings. Social robots, defined as autonomous or semi-autonomous systems capable of interacting with humans in accordance with established social norms (Belpaeme et al., 2018; Neumann, 2020), have been examined in various educational roles, including tutors, peer learners, teaching assistants, and tutees (Belpaeme et al., 2018; Woo et al., 2021). Applications span the instruction of second-language vocabulary, mathematics, and computational thinking, among others (e.g., Derakhshan et al., 2025; Ouyang and Xu, 2024; Papadopoulos et al., 2020).

Proponents of social robots argue that their advantages result from several attributes. A central hypothesis is that physical embodiment, i.e., the ability to gesture, direct gaze, and occupy physical space, enhances student attention and motivation (Donnermann et al., 2020; Lee et al., 2006; Li, 2015). Furthermore, the non-evaluative nature of social robots can mitigate error-related anxiety, creating a safe space for practice, and encouraging help-seeking behaviors (Howley et al., 2014; Wang et al., 2013). These benefits can be augmented through personalization, where robots adapt instructional tactics to individual learners (Baxter et al., 2017; Yang et al., 2024), and by employing novel pedagogical paradigms, such as casting the robot as a tutee to exploit the so-called protégé effect (Tanaka and Matsuzoe, 2012).

Despite the aforementioned optimism, several reservations exist. The literature includes various studies yielding equivocal outcomes, where robotic interventions do not surpass less complex alternatives such as tablets or standard instructional approaches. For example, a study by Zhexenova et al. (2020) compared a robot-only tutor with a tablet-only application and a human-teacher condition for early literacy (letter learning) and found no significant differences in learning gains. Another factor is that the social attributes designed to augment learning may inadvertently introduce distractions. Kennedy et al. (2015b) reported that excessive social behaviors in a robot diminished its effectiveness relative to a nonsocial counterpart. Other studies indicate that engaging robotic elements can divert focus from primary content (Charpentier et al., 2022; Rosenthal-von der Pütten et al., 2016).

Social robots in educational applications also encounter technical limitations. AI elements, such as speech recognition, frequently exhibit inaccuracies in processing inputs from young children or non-native speakers, yielding user dissatisfaction (Banaeian and Gilanlioglu, 2021; Bhat et al., 2016; Liebens, 2019; Randall, 2019). Text-to-speech systems are often critiqued for lacking natural intonation, which may hinder comprehension and learning (Lee et al., 2010). Perceptual deficiencies, such as failures in user detection, compound these issues, often requiring Wizard-of-Oz experiments instead of an actual autonomous deployment of the robot (e.g., Kennedy et al., 2016b; Komatsubara et al., 2014). High costs, content development, and maintenance also present barriers (Papadopoulos et al., 2020). Certain psychological drawbacks further impede the assimilation of social robots. In particular, the novelty effect, although initially increasing motivation, typically fades quickly (Baxter et al., 2017; Kanda et al., 2007).

A source of the conflicting outcomes in the literature may be the robot’s assigned pedagogical function and the chosen control condition. Comparisons against no-intervention controls typically confirm the robots’ instructional viability compared to no training (Draper and Clayton, 1992; Kennedy et al., 2016b; Vogt et al., 2019). Although human tutors frequently elicit greater learning gains (Kennedy et al., 2016a; Zhanatkyzy et al., 2022), robots may excel in constrained contexts such as routine exercises (e.g., Chang and Hwang, 2024). For example, for syntax drills, it has been found that a robotic tutor surpassed human teachers, a result that was attributed to the robot’s inexhaustible consistency (Iio et al., 2024). The paramount evaluation, however, involves contrasts with virtual interfaces such as tablets. Herein, substantiation of the incremental value of social robots remains contentious. Several studies found no learning benefits for embodied robots relative to screen interfaces (e.g., Verhelst et al., 2024; Vogt et al., 2019), while other studies assert that social robots outperform tablets (e.g., Konijn et al., 2022) or that tangible (touch/gesture) interactions are more effective than non-interactive virtual settings (Al Hakim et al., 2022). In summary, the selection of control conditions shapes the robot’s conceptualization as a mere intervention (when compared to nothing), a human co-teacher or surrogate (when compared to a human teacher), or as a sophisticated technology (when compared to a virtual interface).

### The present study: a new meta-analytic synthesis

The wide variation in experimental outcomes requires a new meta-analytic review to synthesize findings and identify the factors that drive learning. Prior meta-analyses have consistently concluded that social robots have a positive overall effect on student learning, for example in language learning (Belpaeme et al., 2018; Derakhshan et al., 2025; Lee and Lee, 2022; Wu and Li, 2024). Some moderators have been identified; for example, effects appear largest for elementary-aged learners (Derakhshan et al., 2025) and elementary/secondary *versus* tertiary school learners (Lee and Lee, 2022; Wu and Li, 2024), and interventions lasting between 1 and 4 weeks produce larger effects than studies with shorter intervention time spans (Wu and Li, 2024).

However, these prior meta-analyses are constrained by limitations, which indicate the need for a more comprehensive and rigorous synthesis. A primary issue is the limited statistical power caused by the small number of included studies, which has prevented powerful moderator analyses. Previous meta-analyses also suffer from methodological inconsistencies, including the aggregation of diverse control conditions, the conflation of cognitive and affective outcomes (Sukardi et al., 2024; Wang K. et al., 2023; Wu and Li, 2024), the inclusion of non-physical chatbots under the “robot” umbrella (Wu and Li, 2024), and the inclusion of effect sizes without a non-robot control group (Derakhshan et al., 2025; Wu and Li, 2024).

The current meta-analysis includes a larger corpus of studies to address these shortcomings. Our primary goal is not to recalculate an overall effect size but to deconstruct it. We focus on disentangling the robot’s effectiveness based on its pedagogical role, with the hypothesis that the distinction between a co-teaching assistant and a full teacher replacement is a primary source of the heterogeneity observed in the literature. Furthermore, this study introduces a novel sentiment analysis using a Large Language Model (LLM). This analysis measures “Positiveness” in research papers to investigate potential reporting biases and examines how this sentiment relates to reported effect sizes and geographical origin to explore whether a tendency toward spin may be distorting the field’s conclusions.

## Methods

Our literature search and reporting adhered to the PRISMA guidelines (Page et al., 2021), see supplementary material. Literature searches were conducted using Google Scholar (latest search date: 13 September 2024). Instead of fixed search strings, we used combinations of keywords related to the robot (e.g., “social robot”, “humanoid robot”, “robot tutor”) and the outcome (e.g., “learning”, “cognitive skills”, “education”, “post-test”). This approach was needed because of the diverse terminology in the field to achieve comprehensive retrieval of relevant studies. This keyword search was supplemented by a manual review of the reference lists of previous meta-analyses and a prior review on social robots (Johal, 2020) to assess whether there were further relevant studies for inclusion. Furthermore, we performed forward citation tracking for eligible studies and targeted searches for other relevant publications by the authors of these studies. Additional searches were conducted through ResearchGate and using Google Search. This iterative process continued until no new eligible studies were being identified.

The selection process was conducted by two of the authors. One author conducted the initial screening and data extraction, with the last author verifying all inclusions and resolving ambiguities. No automation tools were used in the process.

### Included studies

Studies were included if they used a physical social robot for training cognitive skills in human participants. Studies in which the robot was used as an *additional* teaching medium/assistant to a human teacher (e.g., Chang and Hwang, 2024) or to a virtual interface (e.g., Konijn and Hoorn, 2020) were included, as were studies in which the robot was controlled through a Wizard-of-Oz method. Additionally, a number of studies (mostly due to COVID-19 restrictions or inspired by the circumstances at the time) involving the presentation of a physical social robot in video conferencing mode were included (Donnermann et al., 2021; Donnermann et al., 2024; Kanero et al., 2021; Li et al., 2016; Norman et al., 2022; Steinhaeusser et al., 2022; Velentza, 2024a). However, studies without a physical robot but with only a virtual-reality representation of a robot or an animated character (e.g., Davis et al., 2023) were not included. Studies in which participants created or programmed a robot kit (e.g., Lego Robotics) were not included, as these do not concern social robots. Studies in which performance improvement was attempted not through cognitive training but, for example, through emotional training or mindfulness, were also not included (e.g., Tozadore et al., 2023).

To facilitate a broad analysis of how effect sizes vary by study design, we deliberately adopted an inclusive approach to methodological rigor. Consequently, our sample was not limited to strictly randomized controlled trials; we also included studies with variations such as cluster randomization (e.g., by school class) or non-randomized, self-selected groups. Following this principle, studies with brief cognitive assessments were also accepted. Studies with a large discrepancy in pre-test scores between the groups were not included, but were retained for the post-test vs. pre-test analysis (see below).

In the vast majority of cases, the studies used a between-subjects design. However, a small number of studies used a within-subjects design (Bravo Perucho and Alimardani, 2023; De Haas et al., 2020; Hu et al., 2023; Kory Westlund et al., 2017; Movellan et al., 2009; Tanaka and Matsuzoe, 2012). These studies were also included.

We did not include studies on autistic persons (e.g., Alemi et al., 2015; So et al., 2018; Taheri et al., 2016), people with learning disabilities (e.g., Pérez et al., 2021; Reardon et al., 2019), hearing impairments (e.g., Akalın et al., 2014), or dementia (e.g., Feng et al., 2022; Valentí Soler et al., 2015). These groups were excluded because it was expected that their learning abilities differ from the general population and may also involve different types of tasks to be learned.

To be included, the study had to measure learning effectiveness using a test administered after completion of the training, also referred to as a post-test. We excluded studies where test questions were interspersed within the training or where the robot itself asked the test questions, provided hints for solving them, or gave knowledge-of-results feedback on (some of) the test questions (e.g., Chen Hsieh, 2021; Lee and Specht, 2023; Leyzberg et al., 2014; Wedenborn, 2015). In other words, this meta-analysis focuses on comparing learning outcomes under equal post-test conditions and does not address how robots impact task performance.

Additionally, to be included in the analysis, the means and standard deviations of performance scores had to be available. In some cases, these values were extracted from figures in the paper, calculated from raw data presented in the figures, obtained from a data repository, or estimated using computer simulation. We sent approximately 70 emails in total to authors requesting further information. In about 40% of the cases, we received a response, and about two-thirds of those were useful for improving our annotations (see also Derakhshan et al., 2025, who indicated that few authors respond to queries).

Other variables collected included the country where the study was conducted, the presence or absence of a human tutor during the training, the total duration of the training, and the similarity of the pre- and post-experiment assessments/tests, where applicable.

Since this study focuses on cognitive skills, the following types of effects were not included:

- Affective outcomes.
- Self-reported knowledge or perceived knowledge gain rather than knowledge measured through a test (e.g., Ahmad et al., 2020; Serholt et al., 2022).
- Physical and motor performance (e.g., dancing, wayfinding, theater performance, walking speed, rehabilitation; Al Hakim et al., 2020; Benvenuti and Mazzoni, 2020). However, handwriting skills were included. It was reasoned that handwriting is a typical cognitive skill learned in schools.
- Attitude change (e.g., attitudes towards waste recycling; Castellano et al., 2021).
- Social skills, wellbeing, anxiety (e.g., Jeong et al., 2023).

### Effect size calculation–robot group vs. control group

Cohen’s *d* was calculated between the post-test performance of the robot group and the control group. Specifically, for each study, we calculated Cohen’s *d* per score on the outcome measure in the post-test (e.g., number of correctly answered questions, number of answers generated, task completion time) for the robot condition *versus* the control group featuring the same post-test and the same time interval between the training and post-test. In a number of studies (Alves-Oliveira, 2020; Gruson, 2019; Howley et al., 2014; Kennedy et al., 2015a; Mubin et al., 2019; Suzuki and Kanoh, 2015; 2017; Zhanatkyzy et al., 2022), no post-test score was available, but a learning gain relative to a pre-test score was reported; in these cases, we calculated Cohen’s *d* between the learning gains instead of Cohen’s *d* between the post-test scores.

The effect sizes per study were categorized into six different control groups:

1. Audio recording.
2. Human teacher.
3. Nothing (i.e., no training/instruction).
4. Paper (e.g., a list of material to be learned).
5. Sham task, such as interacting with the robot but not engaging with the robot’s educational material (Alves-Oliveira, 2020; Vogt et al., 2019), or engaging in unrelated (cognitive) tasks (Resing et al., 2019; Resing et al., 2020).
6. Virtual interface. This could involve any digital medium, such as interacting with a tablet (e.g., Rintjema et al., 2018; Zhexenova et al., 2020) or laptop (e.g., Miyauchi et al., 2020), but it could also involve a representation of a robot, i.e., a virtual robot (Kennedy et al., 2015a; Rosenthal-von der Pütten et al., 2016).

If, for a given comparison between the robot group and control group, multiple robot groups were available, Cohen’s *d* was weighted according to sample size. Since the effects often involved a (weighted) average of multiple measures (with different standard deviations of the scores) and potentially multiple robot conditions, we used a simplified approach to calculate Cohen’s *d* by assuming equal variance and equal sample size for the robot group and the control group.

Typically, in meta-analyses, either within-study variance (or sample size) determines the weight (fixed-effects meta-analysis), or studies are weighted more equally depending on the degree of between-study variance (random-effects meta-analysis). In determining the overall effect size, we assigned equal weight to all studies, regardless of their sample sizes. However, for studies reporting multiple robot-control comparisons, these were first aggregated internally by weighting them according to their respective sample sizes.

There were a number of reasons for unit-weighting the studies:

1. An exploratory analysis showed substantial between-study variance, with some studies showing a large negative effect of the robot (e.g., Zhanatkyzy et al., 2022) and others a positive effect (e.g., Alemi et al., 2014). This variability could not be explained by sample size alone but rather by the study design.
2. Some studies featured large sample sizes, but should not necessarily be seen as more important. For example, McIntosh et al. (2022) had 995 people interact briefly with a social robot and subsequently answer three health-related questions. Such a study should not necessarily carry more weight than a smaller study where participants interacted with the robot for a longer period or where their skills were assessed in a more comprehensive manner. This idea is consistent with various works indicating that assigning equal weight to effects is sensible, especially when sample sizes vary substantially (Bobko et al., 2007; Osburn and Callender, 1992; Shuster, 2010). Underlying the idea of assigning equal weights to studies is also the notion that sample sizes should not be seen as fixed parameters (as is typically done in meta-analyses) but are themselves subject to random sampling (Shuster, 2010).
3. Using equal weights allowed us to examine the effect of moderator variables in correlational analyses in a simple and transparent manner.

In addition to categorizing studies by control group, we examined two other potential moderator variables to explore the heterogeneity in the robot-versus-control effect sizes. Specifically, we compared the Cohen’s *d* values from studies conducted within *versus* outside Europe. We also investigated the influence of participant age by calculating the correlation between the mean age reported in each study and its corresponding Cohen’s *d*.

### Effect size calculation–post-test vs. pre-test

A large number of studies reported pre-test scores in addition to post-test scores. For explanatory purposes, Cohen’s *d* between the pre-test and post-test was computed, regardless of whether the study featured a control group. For each pre-post effect, we extracted the total duration of the training sessions in minutes (if a range was mentioned, the midpoint was taken). We calculated the Pearson product-moment correlation coefficient between the logarithm of the training duration and the pre-post effect. The logarithmic transformation was used to prevent the data from being overly influenced by a small number of training sessions with very long durations. Additionally, a learning curve typically follows a pattern where performance increases rapidly at first and then levels off as training duration increases, which is another reason to apply the logarithmic transformation.

### Robot effectiveness–text analysis

Recently, it has become possible to summarize and evaluate texts, including entire research papers, using LLMs (e.g., De Winter, 2024; Tabone and De Winter, 2023). Our analysis protocol did not include a component-level assessment for specific internal validity threats (e.g., allocation concealment, participant blinding). Consequently, applying a traditional risk-of-bias tool was not appropriate. Instead, we chose to investigate potential reporting bias across the literature through a supplementary sentiment analysis. Specifically, we used Gemini to analyze the degree of positive wording in the text. Each paper’s text was separately submitted to Google’s Gemini 2.5 Pro (June 2025 version).

We applied a self-consistency method (e.g., Tabone and De Winter, 2023; Tang et al., 2024; Wang X. et al., 2023), in which the same question was posed multiple times with variations in the ordering of items. The average of these outputs was then calculated. The goal of this sentiment analysis was to derive an overall score reflecting the ‘positivity towards the robot’ expressed in each paper.

For each paper, we had Gemini 2.5 Pro answer the following questions:

On a scale of 0–10, …

1. *How positive is the article about the social robot being studied?*
2. *How negative is the article about the social robot being studied?*
3. *How effective is the social robot under evaluation according to the article?*
4. *How ineffective is the social robot under evaluation according to the article?*
5. *How well does the social robot under evaluation perform according to the article?*
6. *How poorly does the social robot under evaluation perform according to the article?*
7. *Based on the introduction, how positive is this article about social robots in general?*
8. *Based on the introduction, how negative is this article about social robots in general?*
9. *Based on the discussion, how elaborately are the limitations of the experiment pointed out?*
10. *Based on the discussion, how elaborately are the strengths of the experiment pointed out?*
11. *To what extent are the authors biased in the positive direction, i.e. overly optimistic about social robots?*
12. *To what extent are the authors biased in the negative direction, i.e., overly skeptical about social robots?*

These 12 items, along with the automatically extracted text from the PDF of the paper, were submitted to the Gemini API. This process was repeated five times, with the 12 items presented in a different random order each time. Subsequently, we calculated a mean score per study for each item.

We then extracted a total score by performing a principal component analysis on the first six items. The six additional items about the paper's introduction, discussion, and potential bias were collected for exploratory purposes (see Supplementary Material). For each included study, a Positiveness score was calculated; across studies, these scores had a mean of 0 and a standard deviation of 1. We analyzed the Positiveness score in a similar manner to how we assessed Cohen’s *d* effect sizes. Additionally, we calculated associations between the Positiveness score and the country of the research publication.

## Results

We retrieved 146 studies, comprising 183 effect sizes between robot group and control group and 372 pre-post effects. Among the 146 studies, effect sizes between the robot and control groups were available for 78 studies, based on 4,819 participants. Pre-post effect sizes were available for 108 studies based on 6,232 participants.

Across the 146 included studies, publications were concentrated in the last decade (2015–2024: 129/146), indicating rapid growth of empirical work on social robots for learning outcomes. Conference proceedings/symposia/workshops (55) and journal articles (76) were represented in high numbers, with the remainder consisting of other formats (15; e.g., theses, reports, preprints).

**FIGURE 1:**
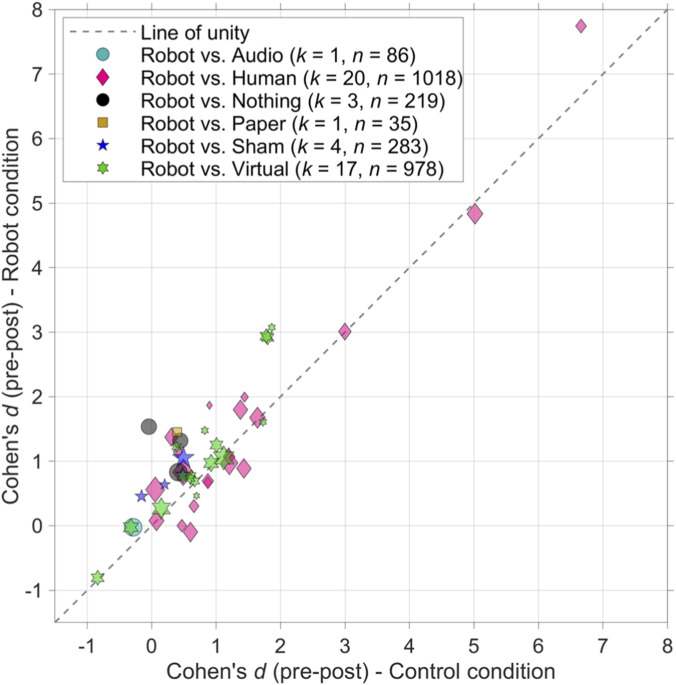
Scatter plot showing the pre-post effect sizes for the robot condition compared to the respective control group. Points above the unity line suggest greater effectiveness in the robot condition relative to the control group. The areas of the markers are linearly related to the sample sizes. *k* = number of studies; *n* = total sample size of the studies combined.

The mean Cohen’s *d* of the 372 pre-post effects was 1.08 (*SD* = 1.32). Figure 1 shows the effects for studies for which a pre-test score and post-test score were available for the robot condition as well as for the control group. It can be seen that in nearly all studies, participants demonstrated learning, indicated by a positive effect (*d* > 0) for both the control group and the robot group.

**FIGURE 2:**
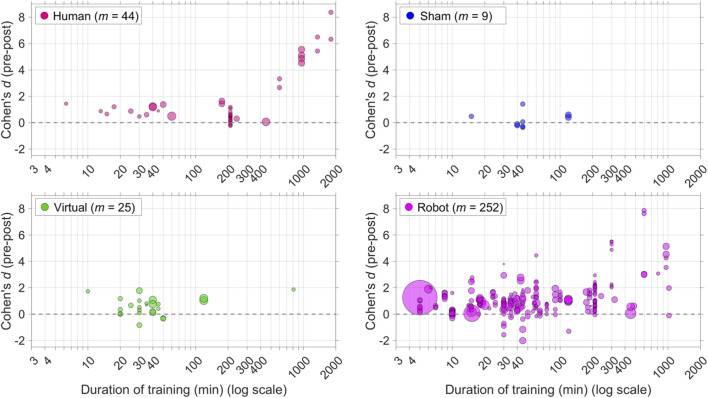
Scatter plots of pre-post effect sizes *versus* the total duration of the training sessions (logarithmic scale). *m* = number of pre-post effects (a study could feature multiple effects). The areas of the markers are linearly related to the sample sizes.

Figure 2 illustrates that the degree of learning relative to the pre-test was higher when the experimental condition featured prolonged training (>500 min). The correlation between training duration and pre-post effects is positive, at *r* = 0.46 (based on *m* = 332 pre-post effects for which the training duration was available; all control conditions combined).

The pre-post effects in Figures 1, 2 are indicative of the amount of learning relative to the pre-test. However, to assess the learning outcome of a social robot, it must be compared with a control group (i.e., the degree to which the marker is above the diagonal in Figure 1).

**FIGURE 3:**
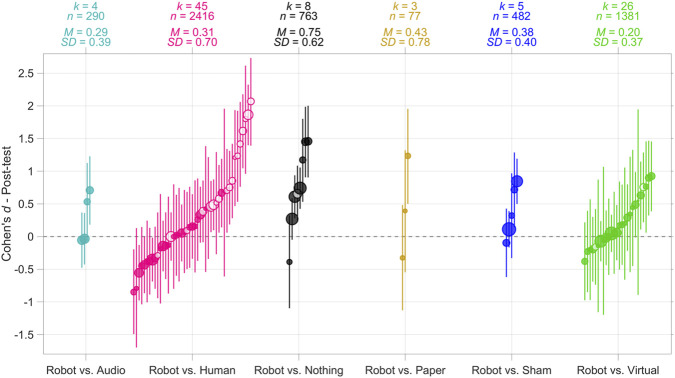
Cohen’s *d* effect sizes for the robot group relative to the control group, for six different types of control groups. *k* = number of studies (note that a single study may contribute to multiple categories if it employed multiple distinct control groups), *n* = total sample size of the studies combined, *M* = mean of all effect sizes (i.e., the overall effect). *SD* = standard deviation of effect sizes. The areas of the markers are linearly related to the sample sizes. The vertical lines represent 95% confidence intervals of Cohen’s *d* assuming equal group sizes for between-subjects comparisons. Markers with a white center refer to studies in which a human tutor was present in the robot condition.

The Cohen’s *d* values for the robot group *versus* the control group are shown in Figure 3, for six types of control groups. Training by means of social robots was found to be more effective than no training (Nothing: *M* = 0.75; *SD* = 0.62; Sham task: *M* = 0.38; *SD* = 0.40). Whether training with a social robot is more effective than training with a virtual interface is less clear (*M* = 0.20; *SD* = 0.37). The comparison between a robot and a human teacher was overall positive (*M* = 0.31; *SD* = 0.70). According to an independent-samples *t*-test (treating comparisons as independent observations), Cohen’s *d* was larger for ‘Nothing’ control groups compared to ‘Virtual interface’ control groups, *t* (32) = 3.03, *p* = 0.005. In other words, compared to no training, the robot appeared more effective than when it was compared with a virtual interface. The difference in Cohen’s *d* between Human control groups and Nothing control groups was not significant, *t* (51) = 1.64, *p* = 0.108.

It can also be observed that, particularly when the control group involved a human teacher, the heterogeneity was high. This heterogeneity can be seen from the high standard deviation of the Cohen’s *d* values (*SD* = 0.70), as well as from the non-overlapping 95% confidence intervals, with some studies showing a negative effect and others a positive effect.

When examining the strongest effects shown in Figure 3, the robot was accompanied by a human teacher (e.g., Tsai, 2019, *d* compared to Human = 2.07; Chang and Hwang, 2024, *d* compared to Human = 1.86; Alemi et al., 2023, *d* compared to Human = 1.80; Wu et al., 2015, *d* compared to Human = 1.62). This is remarkable when considering that in the majority of the studies included in this meta-analysis (59 out of 78, or 76% of studies), the robot was *not* accompanied by a human teacher. The Cohen’s d for robot *versus* human comparisons was larger for studies where the robot was accompanied by a human teacher, i.e., co-teaching (*M* = 0.88; *SD* = 0.68; see 18 white markers in the ‘Robot vs. Human’ column in Figure 3) compared to robot-only conditions (*M* = −0.06; *SD* = 0.40; see 27 solid markers in Figure 3), *t* (43) = 5.83, *p* < 0.001.

**FIGURE 4:**
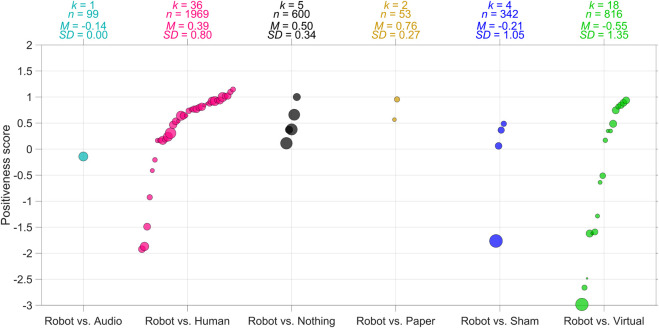
Gemini 2.5 Pro-based Positiveness scores of the research paper for different types of control groups. *k* = number of studies (papers), *n* = total sample size of the studies combined, *M* = mean Positiveness score. *SD* = standard deviation of Positiveness scores. The areas of the markers are linearly related to the sample sizes. Scores are only shown for papers in which only one type of control condition was present. For example, when the paper compared the robot with both a human teacher and a virtual interface, the Positiveness score is not shown.

We repeated the above analysis, this time focusing on the Positiveness scores. The results in Figure 4 show some similarities to those in Figure 3. For instance, the mean Positiveness score for Robot *versus* Nothing papers (*M* = 0.50; *SD* = 0.34) is higher than for Robot *versus* Virtual papers (*M* = −0.55; *SD* = 1.35). However, this difference was not statistically significant, *t* (21) = 1.70, *p* = 0.105.

**FIGURE 5:**
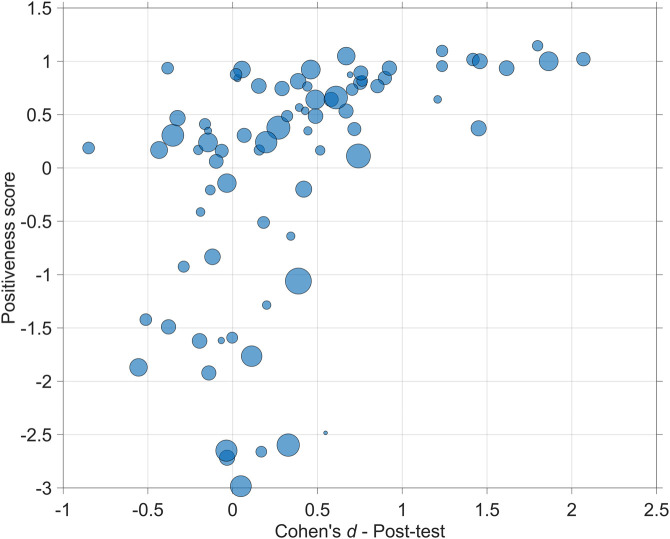
Scatter plot of the Positiveness score *versus* the Cohen’s *d* effect size (*k* = 78). The areas of the markers are linearly related to the sample sizes of the studies.

Additionally, we examined whether the Gemini 2.5 Pro-based scores of the studies were related to the effect sizes as shown in Figure 3. If the robot was compared to more than one control condition (e.g., Human and Nothing, see Rosi et al., 2016), these effects were averaged across these control conditions. Thus, for the correlational analysis, multiple effects within a single study were averaged to produce one effect size per study. It was found that the Positiveness score was positively correlated with effect size (*r* = 0.47, *p* < 0.001, *k* = 78; Spearman’s rank-order correlation = 0.62), as illustrated in Figure 5.

In a further attempt to explain under what conditions positive effect sizes and high Positiveness scores occur, we made a distinction between studies conducted within and outside Europe. This distinction was made after an initial exploration suggested that there might be national differences in the tone of papers, namely papers that seem to promote robots and those that critically evaluate robots.

We found that the effect size of the robot *versus* the control group was weaker for studies from Europe (*M* = 0.17; *SD* = 0.39; *k* = 36) than for studies outside Europe (*M* = 0.53; *SD* = 0.69; *k* = 42), a statistically significant difference, *t* (76) = −2.74, *p* = 0.008. A multivariable linear regression (fitted on 91 study–control-condition pairs) confirmed that this regional difference remained significant (β = −0.37, *p* = 0.005) after controlling for the type of control condition used. Furthermore, a Chi-square test indicated no significant difference in the selection of control conditions between regions (p = 0.105).

Furthermore, it was found that studies from Europe had a lower Positiveness score (*M* = −0.40, *SD* = 1.09, *k* = 65) than studies outside Europe (*M* = 0.32, *SD* = 0.79, *k* = 81), *t* (144) = −4.61, *p* < 0.001. A multivariable regression analysis performed on the subset of studies with a single control condition (*k* = 66) confirmed that this regional disparity remained significant (β = −0.71, *p* = 0.006) after controlling for the control condition type.

**FIGURE 6:**
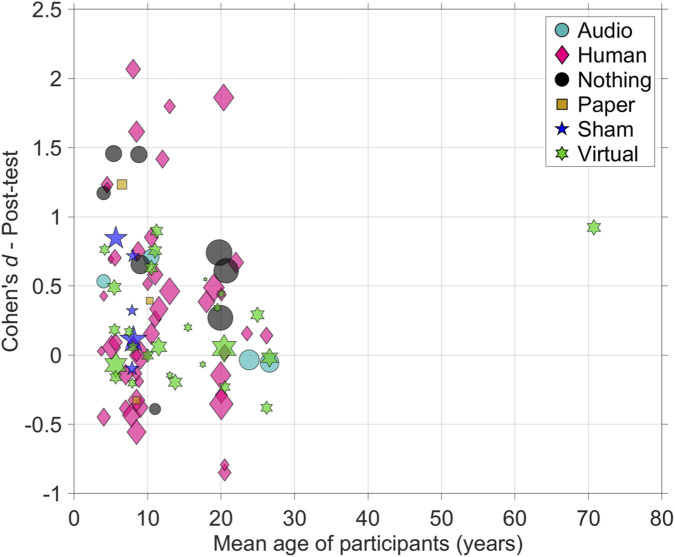
Scatter plot depicting the effect size between social robot and control group *versus* the mean participant age (*k* = 78; 91 effects), stratified by control condition. The areas of the markers are linearly related to the sample sizes of the studies.

In response to the moderation effects that remained unclear in previous meta-analyses, we investigated whether the age of participants was associated with learning outcomes relative to the control group. We found no significant association between age (sample-size-weighted mean participant age in the study) and Cohen’s *d* (*r* = −0.05, *p* = 0.686; Spearman’s rank-order correlation = −0.13, *p* = 0.270, *k* = 78 studies). To determine if age effects varied depending on the control condition, we performed a linear regression analysis with interaction terms (Age × Control Type). The interaction terms were not statistically significant (all *p* > 0.238), indicating that the relationship between age and effect size did not differ across control conditions. Furthermore, examining the groups individually revealed no significant correlations between age and effect size for any control type (e.g., Human: *r* = −0.10, *p* = 0.525; Virtual: *r* = 0.23, *p* = 0.257). Figure 6 presents a scatter plot illustrating the substantial heterogeneity of the effects, where age does not provide an obvious explanatory factor.

## Discussion

This meta-analysis was conducted to synthesize the learning outcomes of educational interventions that used social robots. We focused on how the robot’s pedagogical role and the type of control condition influence effectiveness. Our findings show that the effectiveness of social robots is dependent on their method of implementation. The most important factor is whether the robot is used as a teaching assistant or as a full teacher replacement. In addition, a sentiment analysis suggests that the reported effectiveness of robots may be connected to reporting biases that appear to differ by geographical region.

Our results show that the measured benefit of a social robot is directly dependent on the control condition against which it is compared. The largest effect size appeared when robot interventions were compared to a “nothing” control group (*d* = 0.75). This outcome confirms that a structured educational activity is more effective than no activity.

A central debate in the literature concerns the added value of a robot’s physical embodiment. Our analysis provides evidence to inform this debate, by revealing a modest but positive effect size for robots *versus* virtual interfaces (*d* = 0.20). At first glance, this result suggests that embodiment does confer a learning advantage, and lends support to theories that link physical presence to social presence and attention (Lee et al., 2006; Li, 2015). However, embodiment is not a panacea. Some work even reported that a physical robot hindered learning compared to a virtual agent (Rosenthal-von der Pütten et al., 2016) or that its engaging social features negatively correlated with learning gains, possibly by acting as a distraction that increases cognitive load (Kennedy et al., 2015b). The value of embodiment is likely dependent on task and implementation and not a guaranteed benefit. The type of feedback of the social robot matters; in three of the four studies where the robot was most effective against a virtual interface (*d* > 0.75), the robot offered more interactive and application-focused feedback, i.e., a question-and-answer module or real-life examples (Wei et al., 2021; *d* = 0.92), a variety of interactive functions (Hyun et al., 2008; *d* = 0.76), and more apps (Darmawansah and Hwang, 2024; *d* = 0.76).

This meta-analysis found a positive learning effect for robots *versus* human teachers (*d* = 0.31). The introduction also outlined several mechanisms through which robots might aid learning. One presumed mechanism is the mitigation of anxiety, a concept related to Krashen’s Affective Filter Hypothesis (Hong et al., 2016). Students report feeling less pressure and anxiety with a robot because it is perceived as a non-judgmental partner (Donnermann et al., 2020; Peura et al., 2023; Suzuki and Kanoh, 2017; Wang et al., 2013). One student noted, “*I felt comfortable practicing English with her because she is just a toy and I can say whatever I want with her*” (Wang et al., 2013, p. 306). Some pedagogical strategies deliberately leverage this, programming the robot to make its own mistakes to normalize errors for the students (Alemi and Haeri, 2020; Alemi et al., 2014; Jimenez and Kanoh, 2013). Personalization was also noted as a potential benefit and can be connected to Self-Determination Theory (Lu et al., 2023; Van Minkelen et al., 2020). Our results include a number of studies where personalized robots that adapted to student performance led to better learning outcomes than non-personalized versions (Baxter et al., 2017; Kory-Westlund and Breazeal, 2019; Leyzberg et al., 2018; Park et al., 2019; Ramachandran et al., 2017; Ramachandran et al., 2019b).

The most noteworthy finding of this meta-analysis is the powerful moderating effect of the robot’s pedagogical role. When a robot acts as a teaching assistant in a co-teaching capacity with a human, it produces a strong positive effect on learning (*d* = 0.88). In contrast, when a robot is positioned as a complete replacement for the teacher, it offers no benefit (*d* = −0.06). This result indicates that the most effective use of current social robots in education is as a supportive tool for human teachers, not as a substitute. Current robots often cannot manage classroom dynamics, respond to spontaneous student questions, or handle unforeseen occurrences (Komatsubara et al., 2014; Ponce et al., 2019; Randall, 2019). As interactions can break down, a human may be required to repair these interactions (Bhat et al., 2016; Ponce et al., 2019; Woo et al., 2021). The efficacy of the robot is further constrained by the context gap between the design environment and the deployment environment. Singh et al. (2023) provided evidence from an under-resourced community school where the robot’s Westernized accent forced students to rely on a human moderator for validation. In an assistive role, however, the robot can excel at structured and repetitive tasks. These tasks include leading drills or running quizzes (Alemi et al., 2023; Chang and Hwang, 2024; Wu et al., 2015). This frees the human teacher to provide higher-level support to students (Chang and Hwang, 2024; Wu et al., 2015). This synergy, which combines the robot’s consistency with the human’s adaptability, may be the underlying mechanism that creates an effective learning environment.

Our automated sentiment analysis using a LLM showed that Positiveness scores correlated strongly with Cohen’s *d* effect sizes (*r* = 0.47; Spearman’s rank-order correlation = 0.62). In a further attempt to explain the Positiveness scores, we reasoned that these scores are not just based on the effects found, but possibly also influenced by bias. In other words, the Positiveness scores may be partially attributable to the authors’ inclination to present their findings in a more favorable manner, a practice commonly referred to as ‘spin’ (Boutron et al., 2010). An exploration of extreme effects or extremely high or low Positiveness scores suggests that different authors have different approaches, from introducing a positive spin to applying a critical tone (see Table 1 for an overview). Previous research supports the notion that biases are not isolated but are embedded in the culture of the research team, which can, in part, be influenced by the department, university (Köse and Korkmaz, 2019), or nationality (Vickers et al., 1998; Zhang et al., 2011). While our sentiment analysis attempted to quantify ‘spin’, publication bias is another threat. Although we retrieved many null results, the tendency toward positive sentiment, as shown in Table 1, suggests that successful or positively framed studies may be overrepresented in the present meta-analysis.

**TABLE 1:** Selected references with extreme scores on effect sizes and Positiveness scores.

References	Country	Robot vs. control effect size (rank out of 78)	Positiveness score (rank out of 146)	Selected quote from the paper	Our evaluation
Suzuki and Kanoh (2015)	Japan	*d* = −0.85 (1st, lowest overall)	0.19 (60th)	“*The results show that the robot provides the same effectiveness when engaging in expression education as does a human teacher*” (p. 498)	The authors claim equivalence despite a large (or inconclusive) negative effect size.
Alemi et al. (2023)	Iran	*d* = 1.80 (76th)	1.15 (146th, highest overall)	“*RALL can be a very appropriate alternative needing more attention by policymakers and language practitioners*” (p. 13)	The extremely positive sentiment score is consistent with the large effect size (*d* = 1.80) reported in the study.
Riedmann et al. (2024)	Germany	*d* = 0.05 (28th)	−2.98 (1st, lowest overall)	“*The generally high performance in test and post-test might be an indication of a ceiling effect with the potential problem to allow no sufficient differentiation between individual learners” (p. 381), “Due to the absence of desired effects, it is questionable to what extent the social robot was suitable for the implemented learning scenario. Even though …participants in the present study even reported the robot as an interesting and fun learning partner, there was no discernible benefit*” (p. 382)	The authors candidly acknowledge the null result and explicitly question the utility of the robot.
Van den Berghe (2019)	Netherlands	*d* = −0.03 (24th)	−2.72 (2nd)	“*We tried to stimulate the interaction between the robot and the child … but perhaps more interaction is required … the robot was controlled* via *an assistant in real time, which entailed that its responses were rather slow*” (p. 121)	The tone is critical, aligning the null learning result with a transparent discussion of technical limitations (e.g., slow response times).
Bravo Perucho and Alimardani (2023)	Netherlands	*d* = 0.55 (54th)	−2.48 (7th)	“*students reported a significantly lower impression of robot’s usefulness for their activity”* (p. 358)	Despite finding a moderate positive learning effect, the authors focus the narrative on negative user perceptions.
Chandra et al. (2015)	Portugal/Switzerland	*d* = −0.38 (5th)	0.94 (134th)	“*The results of the study suggest that the teacher-children felt more responsible over the learner-children’s performance in the presence of robot facilitator*” (p. 172).	The abstract mentions positive social outcomes (responsibility) while not mentioning the null result in the abstract.

Finally, our analysis found no reliable relationship between participant age and learning effect sizes, a finding that remained consistent also when disaggregating the data by control condition. The wide scatter of data visible in our analysis (Figure 6) suggests that factors such as the robot’s specific role, the nature of the learning task, and the quality of the instructional design are more powerful predictors of success than the learner’s age alone.

## Conclusion and recommendations

This meta-analysis shows that social robots can be effective educational tools, but it also qualifies this finding: the effectiveness of social robots is not a property of the technology but a function of their pedagogical implementation. Robots acting as assistants in a co-teaching relationship with a human educator produce large learning gains, while robots positioned as replacements for teachers are ineffective. Therefore, this meta-analysis suggests that the most promising path forward is not to develop robots that can supplant human teachers, but rather to design them as tools that support human-led instruction.

This study has several limitations. Our broad inclusion criteria allowed for a broad analysis but means that the overall effect sizes are averaged across studies of varying quality. The decision to weight all studies equally, although justified by the heterogeneity of designs and sample sizes, is a departure from traditional meta-analytic practice. Additionally, the sentiment analysis, while novel, is an exploratory method for investigating reporting bias and should be interpreted with caution. Finally, because we excluded affective outcomes, our findings apply only to cognitive performance. A robot that fails to improve test scores may still offer value through mechanisms not measured here.

In conclusion, the debate on social robotics should not focus on whether robots can teach but on *how* they can best be used to support learning. For practitioners, this meta-analysis provides a clear recommendation: social robots should be used as assistive tools to enhance, not replace, human instruction. As AI capabilities advance, the robot may eventually be able to function as a complete substitute for a human teacher; however, being able to replace a teacher does not mean one should. Future research must guide this transition based on learning outcomes, rather than technological availability alone.

## Data Availability

The data and analysis scripts supporting the findings of this study are available online (https://doi.org/10.4121/a78b6b99-3fdf-4ae0-97b0-3b618b00805e), including the coded study data, Gemini-based sentiment scores, and MATLAB scripts for reproducing all analyses and figures.
